# Clinical and microbiological features of positive blood culture episodes caused by non-fermenting gram-negative bacilli other than *Pseudomonas* and *Acinetobacter* species (2020–2023)

**DOI:** 10.1007/s15010-024-02342-6

**Published:** 2024-07-11

**Authors:** Roberto Casale, Matteo Boattini, Sara Comini, Paulo Bastos, Silvia Corcione, Francesco Giuseppe De Rosa, Gabriele Bianco, Cristina Costa

**Affiliations:** 1 Department of Public Health and Paediatrics, University of Turin, Turin, Italy; 2 Microbiology and Virology Unit, University Hospital Città della Salute e della Scienza di Torino, Corso Bramante 88/90, Turin, 10126 Italy; 3 Lisbon Academic Medical Centre, Lisbon, Portugal; 4 Operative Unit of Clinical Pathology, Carlo Urbani Hospital, Jesi, 60035 Italy; 5 Department of Medical and Clinical Pharmacology, Toulouse University Hospital, Toulouse, France; 6 Department of Medical Sciences, Infectious Diseases, University of Turin, Turin, 10124 Italy; 7 Unit of Infectious Diseases, Cardinal Massaia Hospital, Asti, 14100 Italy; 8 Department of Experimental Medicine, University of Salento, Via Provinciale Monteroni n. 165, Lecce, 73100 Italy

**Keywords:** Non-fermenters, Bloodstream infection, Sepsis, Gram-negative bacilli, *Stenotrophomonas*, *Achromobacter*, *Burkholderia pseudomallei*, Melioidosis

## Abstract

**Introduction:**

Non-fermenting Gram-negative bacilli (NFGNB) other than *Pseudomonas aeruginosa* and *Acinetobacter baumannii* complex are pathogens of interest due to their ability to cause health-care associated infections and display complex drug resistance phenotypes. However, their clinical and microbiological landscape is still poorly characterized.

**Methods:**

Observational retrospective study including all hospitalized patients presenting with a positive positive blood culture (BC) episode caused by less common NFGNB over a four-year period (January 2020–December 2023). Clinical-microbiological features and factors associated with mortality were investigated.

**Results:**

Sixty-six less common NFGNB isolates other than *Pseudomonas* and *Acinetobacter* species causing 63 positive BC episodes were recovered from 60 patients. Positive BC episodes were predominantly sustained by *Stenotrophomonas maltophilia* (49.2%) followed by *Achromobacter* species (15.9%) that exhibited the most complex resistance phenotype. Positive BC episodes had bloodstream infection criteria in 95.2% of cases (60 out 63), being intravascular device (30.2%) and respiratory tract (19.1%) the main sources of infection. Fourteen-day, 30-day, and in-hospital mortality rates were 6.4%, 9.5%, and 15.9%, respectively. The longer time from admission to the positive BC episode, older age, diabetes, admission due to sepsis, and higher Charlson Comorbidity Index were identified as the main predictors of in-hospital mortality.

**Conclusions:**

Positive BC episodes sustained by NFGNB other than *Pseudomonas* and *Acinetobacter* species were predominantly sustained by *Stenotrophomonas maltophilia* and *Achromobacter* species, having bloodstream infection criteria in the vast majority of cases. Factors that have emerged to be associated with mortality highlighted how these species may have more room in prolonged hospitalisation and at the end of life for patients with chronic organ diseases.

## Introduction

Gram-negative bacteria bloodstream infection (BSI) is a serious clinical condition associated with notable mortality and morbidity rates, especially among immunocompromised and elderly patients [1, 2]. The course of infection is heavily influenced by various microbiological and clinical issues as the type of pathogen and burden of drug resistance [3], patients conditions and rapidity and appropriateness of diagnosis and treatment [4, 5]. Among non-fermenting Gram-negative bacilli (NFGNB) [6], *Pseudomonas aeruginosa* and *Acinetobacter baumannii* complex are the most frequently isolated pathogens [1, 2]. Given their high burden of antimicrobial resistance [7] they have been placed on the World Health Organisation’s critical priority list for the development of new antibiotics [8]. These two species have therefore been the target of several surveillance [9], clinical [10–12] and microbiological studies [13–15]. Interest in them even increased during the SARS-CoV-2 pandemic due to their frequent detection especially in patients with prolonged time of invasive ventilation and hospitalisation [16, 17]. Among NFGNB, however, there are other bacteria such as *Stenotrophomonas*, *Achromobacter*, *Ochrobactrum*,* Burkholderia*, *Aeromonas*, *Roseomonas*, *Elizabethkingia*, *Chryseobacterium*, *Alcaligenes*, *Ralstonia*, *Cupriavidus*, *Sphingomonas*, *Rhizobium*, *Empedobacter*, *Brevundimonas* and other species that are less frequently diagnosed but of interest due to their ability to display complex drug resistance phenotypes [18–23]. These organisms are widely distributed in the environment, thriving in soil and water supplies [18]. They initially aroused attention due to their ability to colonise and infect cystic fibrosis [24–26] and chronic lung disease patients [27]. They have then emerged as healthcare-associated opportunistic pathogens [28, 29], mainly causing pneumonia and/or BSI [6] and nosocomial outbreaks [30–33], relying on their ability of colonizing medical devices and therapeutic equipment [31, 33].

Risk factors for infection include older age, malignancy, immunodepression, chronic respiratory disease, presence of intravascular device or drain tube, prolonged antibiotic use and hospital length of stay. *Stenotrophomonas maltophilia* is the most detected aetiology [6]. The mortality rate of infections caused by these species is reported to vary greatly depending on the type of infection, pathogen, and patient involved [6].

In recent years, the diagnostic accuracy and frequency of diagnosis of NFGNB other than *Pseudomonas* and *Acinetobacter* species have certainly increased, but their pathogenic potential remains a topic yet to be explored. The clinical significance of these species detection from blood cultures (BCs) sometimes can be unclear, especially in case of polymicrobial infections or with a low positive/overall BC samples ratio. However, the treatment of associated infections presents a real challenge, as these organisms, which are difficult to eradicate from healthcare facilities, often exhibit a complex resistance phenotype through the production of β-lactamases and mechanisms favouring drug efflux [18, 34]. This might result in a high rate of empirical therapy misuse and widespread antibiotic consumption.

EUCAST has also recently introduced clinical breakpoints for *Stenotrophomonas maltophilia*, *Aeromonas* and *Achromobacter* species [35], but therapeutic recommendations of the associated infections are only partially present in the most widely consulted guidelines [36].

We conducted this four-year monocentric study with the objective of investigating the factors associated with mortality in a cohort of patients who presented with a positive BC episode caused by NFGNB other than *Pseudomonas* and *Acinetobacter* species, contributing to the clinical and microbiological characterization of these infections.

## Methods

### Study design

In this observational retrospective four-year study (January 2020–December 2023), we included all NFGNB isolates other than *Pseudomonas* and *Acinetobacter* species recovered from positive BCs of patients admitted at the “Città della Scienza e della Salute di Torino”, a 1900-bed tertiary referral hospital in Turin, Italy. Duplicate isolates obtained within a 20-day interval from the same patient were considered as part of a single positive BC episode and therefore excluded from the analysis. The electronic medical records of patients who presented with a positive BC episode caused by NFGNB isolates other than *Pseudomonas* and *Acinetobacter* species were retrospectively reviewed. Clinical and microbiological characteristics, and factors associated with in-hospital mortality were investigated.

### Microbiological diagnostics

The BACT/ALERT FA and FN Plus BC bottles (bioMérieux, Marcy l’Ètoile, France) were incubated in the BACT/ALERT Virtuo (bioMérieux, Marcy l’Ètoile, France). Positive BCs were subjected to Gram staining and subculture on MacConkey and Blood agar media. Bacterial species identification was performed on overnight subcultures by using matrix-assisted laser desorption ionization–time of flight mass spectrometry (MALDI-TOF MS, Bruker DALTONIK GmbH, Bre-men, Germany). Antimicrobial susceptibility testing was performed through a micro-dilution method (Panel NMDR on automated Microscan WalkAway 96 Plus System, Beckman Coulter, Nyon, Switzerland). Cefiderocol susceptibility testing was carried out by disc diffusion method. Antimicrobial susceptibility testing results were interpreted according to the current EUCAST clinical breakpoints (v. 14.0) [35, 37].

### Statistical analysis

Descriptive data are shown as relative (%) and absolute (n) frequencies for categorical data and median and interquartile range (IQR) for continuous variables. χ2 test or Fisher’s exact test were employed to assess the association between binary variables and outcomes. For continuous variables, the Mann-Whitney U test was used to com-pare distributions between groups based on outcome status. XGBoost was used as an explanatory model to rank and identify relative feature importance, using SHAP values as a proxy for feature importance measured at the local data point level. SHAP values are reported as log odds. Analyses have been carried out in Python 3.10.

## Results

Sixty-six NFGNB isolates other than *Pseudomonas* and *Acinetobacter* species causing 63 positive BC episodes (overall prevalence of 1.6% among Gram-negative bacteria) were recovered from 60 patients. Patients had a median age of 65 years [51.5–74.5], were predominantly male (60.3%) and admitted in medical wards (46%), with a median Charlson comorbidity index estimating a 53% probability of surviving in the next ten years (Table 1).

**Table 1:** Clinical features of patients who presented with a positive blood culture episode sustained by non-fermenting Gram-negative bacilli other than *Pseudomonas* and *Acinetobacter* species (*n* = 63)

Feature	Median [IQR]	% (*n*)
*Patient characteristics*		
Age	65.0 [51.5–74.5]	∴
Male gender	∴	60.3 (38)
Medical	∴	46 (29)
Surgical	∴	22.2 (14)
Critically ill	∴	31.8 (20)
Diabetes	∴	20.6 (13)
Cardiovascular disease	∴	52.4 (33)
Chronic kidney disease	∴	15.9 (10)
Chronic respiratory disease	∴	12.7 (8)
Chronic liver disease	∴	12.7 (8)
Solid neoplasm	∴	17.5 (11)
Hematological neoplasm	∴	17.5 (11)
Solid organ transplant recipient	∴	9.5 (6)
Hematopoietic stem-cell transplantation	∴	4.8 (3)
Immunosuppressive therapy	∴	20.6 (13)
Long-term intravascular device	∴	71.4 (45)
Charlson Comorbidity Index	4.0 [2.5-6.0]	∴
Rectal colonization by multidrug resistant organism	∴	38.1 (24)
Antibiotic therapy in the previous seven days	∴	47.6 (30)
*Microbiological findings*		
Time from admission to positive blood culture episode (days)	17.0 [2.0-34.5]	∴
At least 50% of blood cultures positive	∴	68.3 (43)
*Stenotrophomonas maltophilia*	∴	49.2 (31)
*Achromobacter* species	∴	15.9 (10)
*Ochrobactrum* species	∴	9.5 (6)
*Aeromonas* species	∴	9.5 (6)
*Burkholderia* species	∴	7.9 (5)
Other	∴	14.3 (9)
Polymicrobial blood culture	∴	41.3 (26)
*Reason for admission*		
Trauma	∴	20.6 (13)
Respiratory failure	∴	6.4 (4)
Cardiovascular failure	∴	4.8 (3)
Liver failure	∴	4.8 (3)
Renal failure	∴	1.6 (1)
Sepsis	∴	27 (17)
Surgery	∴	25.4 (16)
Burn injury	∴	6.4 (4)
Stroke	∴	3.2 (2)
*Source of infection*		
Catheter-related	∴	30.2 (19)
Primary	∴	17.5 (11)
Respiratory tract	∴	19.1 (12)
Urinary tract	∴	1.6 (1)
Intra-abdominal	∴	15.9 (10)
Soft tissue	∴	11.1 (7)
*Severity of bloodstream infection*		
ICU admission	∴	14.3 (9)
Pitt Bacteremia Score	1.0 [0.0–2.0]	∴
Neutropenia	∴	9.5 (6)
*Management*		
No treatment	∴	4.8 (3)
Active antibiotic therapy within 48 h	∴	55.6 (35)
*Targeted antibacterial therapy*		
Monotherapy	∴	47.6 (30)
Combination therapy	∴	47.6 (30)
Third-generation cephalosporin-including	∴	9.5 (6)
Cefepime-including	∴	6.4 (4)
Ceftobiprole-including	∴	1.6 (1)
Cefiderocol-including	∴	3.2 (2)
Piperacillin/tazobactam-including	∴	23.8 (15)
Ceftolozane/tazobactam-including	∴	1.6 (1)
Ceftazidime/avibactam-including	∴	7.9 (5)
Meropenem-including	∴	25.4 (16)
Meropenem/vaborbactam-including	∴	1.6 (1)
Fosfomycin-including	∴	6.4 (4)
Aminoglycoside-including	∴	11.1 (7)
Fluoroquinolone-including	∴	22.2 (14)
Tetracycline-including	∴	4.8 (3)
Colistin-including	∴	4.8 (3)
Trimethoprim/sulfamethoxazole-including	∴	17.5 (11)
*Outcomes and complications*		
Relapse of positive blood culture episode at least 14 days after the first	∴	6.4 (4)
Length of stay (days)	41.0 [19.5–72.0]	∴
14-day mortality	∴	6.4 (4)
30-day mortality	∴	9.5 (6)
In-hospital mortality	∴	15.9 (10)

*Abbreviations* IQR: interquartile range; ICU: Intensive Care Unit

**Table 2:** Antimicrobial susceptibility profiles of non-fermenting gram-negative bacilli other than *Pseudomonas* and *Acinetobacter* species included in the study according EUCAST guidelines (v. 14.0)

	*Stenotrophomonas maltophilia* (*n* = 31)		*Achromobacter* species^1^ (*n* = 10)		*Aeromonas* species^2^ (*n* = 6)		*Ochrobactrum anthropi* (*n* = 6)		*Burkholderia species*^3^ (*n* = 5)		Other^4^ (*n* = 9)	
	MIC Range (mg/L)	Resistance† % (*n*)	MIC Range (mg/L)	Resistance† % (*n*)	MIC Range (mg/L)	Resistance† % (*n*)	MIC Range (mg/L)	Resistance† % (*n*)	MIC Range (mg/L)	Resistance† % (*n*)	MIC Range (mg/L)	Resistance† % (*n*)
**PTZ**	≤ 8 to > 16	82.6 (19/23)	≤ 8 to > 16	100 (2/2)	≤ 8 to > 16	16.7 (1/6)	≤ 8 to > 16	66.6 (4/6)	≤ 8 to > 16	50 (2/4)	≤ 8 to > 16	42.9 (3/7)
**CTZ**	≤ 1 to > 4	-	4 to > 8	-	≤ 1	-	4 to > 4	-	≤ 1 to > 4	-	≤ 1 to > 4	-
**CZA**	≤ 2 to > 8	-	≤ 2 to > 8	-	≤ 2	-	≤ 2 to > 8	-	≤ 2 to 4	-	≤ 2 to > 8	-
**CTX**	≤ 1 to > 32	100 (22/22)	32 to > 32	100 (4/4)	≤ 1	-	8 to > 32	100 (4/4)	16 to > 32	100 (4/4)	≤ 1 to > 32	75 (3/4)
**CAZ**	≤ 1 to > 32	71.4 (15/21)	≤ 1 to > 32	70 (7/10)	≤ 1	0 (0/6)	8 to > 32	100 (6/6)	2 to 16	60 (3/5)	2 to > 32	57.1 (4/7)
**FEP**	≤ 0.5 to > 8	-	8 to > 8	-	≤ 0.5	0 (0/6)	2 to > 8	-	4 to > 8	-	≤ 0.5 to > 8	-
CFDC*	30–34 mm	0 (0/4)	35–39 mm	-	-	-	18 mm	-	36–40 mm	-	-	-
**IPM**	≤ 1 to > 8	86.4 (19/22)	≤ 1 to > 8	40 (4/10)	≤ 1 to 4	33.3 (2/6)	≤ 1 to 2	0 (0/6)	≤ 1 to 8	50 (2/4)	≤ 1 to > 8	42.9 (3/7)
**MEM**	≤ 0.12 to > 32	86.4 (19/22)	≤ 0.12 to > 32	30 (3/10)	≤ 0.12 to 4	16.7 (1/6)	1	0 (0/6)	1 to 2	0 (0/5)	≤ 0.12 to 32	42.9 (3/7)
**ERT**	≤ 0.12 to > 1	-	≤ 0.12 to 0.5	-	≤ 0.12 to > 1	-	≤ 0.12 to 1	-	> 1	-	≤ 0.12 to > 1	-
**AK**	≤ 8 to > 16	-	16 to > 16	-	≤ 8 to 16	-	≤ 8 to 16	-	16 to > 16	-	≤ 8 to > 16	-
**GM**	≤ 2 to > 4	-	> 4	-	≤ 2 to 4	-	≤ 2 to > 4	-	> 4	-	≤ 2 to > 4	-
**CIP**	≤ 0.06 to > 1	82.8 (24/29)	1 to > 1	90 (9/10)	≤ 0.06	0 (0/6)	≤ 0.06 to > 1	50 (3/6)	0.5 to 1	100 (4/4)	≤ 0.06 to 0.5	28.6 (2/7)
**LVX**	≤ 0.5 to > 1	24.1 (7/29)	≤ 0.5 to > 2	80 (8/10)	≤ 0.5	0 (0/6)	≤ 0.5 to > 1	33.3 (2/6)	≤ 0.5 to > 1	80 (4/5)	≤ 0.5 to 1	14.3 (1/7)
**CL**	≤ 2 to > 4	-	≤ 2 to > 4	-	≤ 2	-	≤ 2 to > 4	-	> 4	-	≤ 2 to > 4	-
**FF**	≤ 16 to > 64	-	> 64	-	≤ 16 to 64	-	> 64	-	> 64	-	≤ 16 to > 64	-
**TMP/SMX**	≤ 2 to > 4	3.2 (1/31)	≤ 2	-	≤ 2	0 (0/6)	≤ 2	-	≤ 2	0 (0/1)	≤ 2 to > 4	-
**TIGE**	≤ 1 to > 2	-	≤ 1 to 4	-	≤ 1	-	≤ 1 to > 2	-	≤ 1 to 2	-	≤ 1	-

^1^*Achromobacter xylosoxidans* (*n* = 9), *Achromobacter insolitus* (*n* = 1); ^2^*Aeromonas caviae* (*n* = 3), *Aeromonas veronii* (*n* = 2), *Aeromonas bestiarum* (*n* = 1); ^3^*Burkholderia cepacia* (*n* = 2), *Burkholderia vietnamiensis* (*n* = 2), *Burkholderia pseudomallei* (*n* = 1); ^4^*Rhizobium radiobacter* (*n* = 3), *Chryseobacterium arthrosphaerae* (*n* = 2), *Roseomonas mucosa* (*n* = 1), *Empedobacter falsenii *(*n* = 1), *Elizabethkingia anophelis* (*n* = 1), *Brevundimonas diminuita* (*n* = 1)

† Resistance or the MIC value suggesting that the agent should not be used for therapy

* Cefiderocol susceptibility was tested by disc diffusion method

Abbreviations PTZ: piperacillin/tazobactam; CTZ: ceftolozane/tazobactam; CZA: ceftazidime/avibactam; CTX: cefotaxime; CAZ: ceftazidime; FEP: cefepime; CFDC: cefiderocol; IPM: imipenem; MEM: meropenem; ERT: ertapenem; AK: amikacin; GM: gentamicin; CIP: ciprofloxacin; LVX: levofloxacin; CL: colistin; FF: fosfomycin; TMP/SMX: trimethoprim/sulfamethoxazole; TIGE: tigecycline

Among the characteristics of the patients investigated, presence of a long-term intravascular device (52.4%) and cardiovascular disease (71.4%) followed by having been treated with antibiotic therapy in the previous seven days (47.6%), being colonized by multidrug resistant bacteria (28.6%), and neoplasms (35%) were the most prevalent. Patients were predominantly admitted due to sepsis (27%), surgery (25.4%), and trauma (20.6%). The median time to positive BC episode from admission was 17 days [2–34.5] and 68.3% of patients had at least 50% positive BC bottles out of the total number of BC bottles processed. Almost half (41.3%, *n* = 26) of the BC samples were polymicrobial mainly with other Gram-negative bacteria (57.7%, *n* = 15). Positive BC episodes were predominantly sustained by *Stenotrophomonas maltophilia* (49.2%) followed by *Achromobacter* (15.9%), *Ochrobactrum* (9.5%), *Aeromonas* (9.5%), and *Burkholderia* species (7.9%, with one imported case of melioidosis). Other species accounted for 14.3% and included *Rhizobium radiobacter*, *Chryseobacterium arthrosphaerae*, *Roseomonas mucosa*, *Empedobacter falsenii*, *Elizabethkingia anophelis*, and *Brevundimonas diminuita*. Positive BC episodes had BSI criteria in 95.2% of cases (60 out 63), being intravascular device (30.2%) and respiratory tract (19.1%) the main sources of infection. Patients suffered from BSI had a median Pitt Bacteremia score of one [0–2], presented with neutropenia in 9.5%, and required ICU admission in 14.3% of the cases. The majority of patients (55.6%) were treated with active antibiotic therapy within 48 h from positive BC episode detection. Targeted antibacterial therapy was carried out in equal proportions between monotherapy and combination (47.6%). The most frequently used antibiotic regimes were those that included meropenem (25.4%), piperacillin/tazobactam (23.8%), fluoroquinolones (22.2%), and trimethoprim/sulfamethoxazole (17.5%). Regarding complications, four patients (6.4%) reoccurred with a positive BC episode caused by the same organism at least 14 days after the first one. Regarding outcomes, 14-day, 30-day, and in-hospital mortality rates were 6.4%, 9.5%, and 15.9%, respectively. The median length of stay was 41 days [19.5–72].

Antimicrobial susceptibility profiles of NFGNB isolates other than *Pseudomonas* and *Acinetobacter* species included in the study were reported in Table 2. *Stenotrophomonas maltophilia* isolates displayed over 90% susceptibility only to cefiderocol and trimethoprim/sulfamethoxazole. *Achromobacter* species showed high rates of resistance towards all the antibiotics tested and high values of inhibition zone diameter with cefiderocol (range 35–39 mm). *Aeromonas* species displayed susceptibility to third- and fourth-generation cephalosporins, fluoroquinolones and trimethoprim/sulfamethoxazole. *Ochrobactrum anthropi* displayed high susceptibility to carbapenems as well as to meropenem in *Burkholderia* species. High values of the diameter of the zone of inhibition with cefiderocol (range 36–40 mm) were also observed in *Burkholderia* species.

Patients admitted due to trauma had more episodes of positive BC sustained by at least ≥ 50% of the BC bottles positive (27.9% vs. 5%, *p* = 0.047, Table 3). Less frequent species included in the ‘other’ group were more detected in patients with less than 50% of positive BC bottles (30% vs. 7%, *p* = 0.023). No more statistically significant differences in terms of patient characteristics, microbiological findings, source of infection, severity of bacteremia, antibiotic management and outcomes were observed.

**Table 3:** Comparison of the clinical characteristics of patients who had a positive blood culture episode sustained by non-fermenting Gram-negative bacilli other than *Pseudomonas* and *Acinetobacter* species in at least ≥ 50% of blood culture samples sent for microbiological investigation vs. those who had not

Feature	≥ 50% of blood culture bottles positive *n* = 43		< 50% of blood culture bottles positive *n* = 20		*p*-value
	Median [IQR]	% (*n*)	Median [IQR]	% (*n*)	Fisher’s exact/ Mann-Whitney
*Patient characteristics*					
Age	65.0 [47.0–75.0]	∴	64.5 [52.3–72.5]	∴	0.865
Male gender	∴	58.1 (25)	∴	65 (13)	0.783
Medical	∴	48.8 (21)	∴	40 (8)	0.593
Surgical	∴	18.6 (8)	∴	30 (6)	0.342
Critically ill	∴	32.6 (14)	∴	30 (6)	1.000
Diabetes	∴	18.6 (8)	∴	25 (5)	0.739
Cardiovascular disease	∴	55.8 (24)	∴	45 (9)	0.589
Chronic kidney disease	∴	18.6 (8)	∴	10 (2)	0.481
Chronic respiratory disease	∴	16.3 (7)	∴	5 (1)	0.418
Chronic liver disease	∴	11.6 (5)	∴	15 (3)	0.701
Solid neoplasm	∴	14 (6)	∴	25 (5)	0.304
Hematological neoplasm	∴	16.3 (7)	∴	20 (4)	0.732
Solid organ transplant recipient	∴	11.6 (5)	∴	5 (1)	0.655
Hematopoietic stem-cell transplantation	∴	2.3 (1)	∴	10 (2)	0.234
Immunosuppressive therapy	∴	18.6 (8)	∴	25 (5)	0.739
Long-term intravascular device	∴	72.1 (31)	∴	70 (14)	1.000
Charlson Comorbidity Index	4.0 [3.0–6.0]	∴	3.5 [2.0–5.0]	∴	0.507
Rectal colonization by multidrug resistant organism	∴	44.2 (19)	∴	25 (5)	0.173
Antibiotic therapy in the previous seven days	∴	55.8 (24)	∴	30 (6)	0.065
*Microbiological findings*					
Time from admission to positive blood culture episode (days)	17.0 [4.5–37.5]	∴	7.0 [2.0-29.5]	∴	0.395
*Stenotrophomonas maltophilia*	∴	48.8 (21)	∴	50 (10)	1.000
*Achromobacter* species	∴	16.3 (7)	∴	15 (3)	1.000
*Ochrobactrum* species	∴	14 (6)	∴	0 (0)	0.164
*Aeromonas* species	∴	14 (6)	∴	0 (0)	0.164
*Burkholderia* species	∴	7 (3)	∴	10 (2)	0.649
Other	∴	7 (3)	∴	30 (6)	0.023 *
Polymicrobial blood culture	∴	44.2 (19)	∴	35 (7)	0.587
*Reason for admission*					
Trauma	∴	27.9 (12)	∴	5 (1)	0.047 *
Respiratory failure	∴	4.7 (2)	∴	10 (2)	0.586
Cardiovascular failure	∴	2.3 (1)	∴	10 (2)	0.234
Liver failure	∴	4.7 (2)	∴	5 (1)	1.000
Renal failure	∴	2.3 (1)	∴	0 (0)	1.000
Sepsis	∴	27.9 (12)	∴	25 (5)	1.000
Surgery	∴	20.9 (9)	∴	35 (7)	0.351
Burn injury	∴	2.3 (1)	∴	15 (3)	0.090
Stroke	∴	2.3 (1)	∴	5 (1)	0.538
*Source of infection*					
Catheter-related	∴	27.9 (12)	∴	35 (7)	0.571
Primary	∴	11.6 (5)	∴	30 (6)	0.088
Respiratory tract	∴	25.6 (11)	∴	5 (1)	0.083
Urinary tract	∴	2.3 (1)	∴	0 (0)	1.000
Intra-abdominal	∴	16.3 (7)	∴	15 (3)	1.000
Soft tissue	∴	9.3 (4)	∴	15 (3)	0.669
*Severity of bloodstream infection*					
ICU admission	∴	16.3 (7)	∴	10 (2)	0.706
Pitt Bacteremia Score	1.0 [0.0–2.0]	∴	1.0 [0.0–2.0]	∴	0.245
Neutropenia	∴	7 (3)	∴	15 (3)	0.372
*Management*					
No treatment	∴	7 (3)	∴	0 (0)	0.546
Active antibiotic therapy within 48 h	∴	46.5 (20)	∴	75 (15)	0.056
*Targeted antibacterial therapy*					
Monotherapy	∴	53.5 (23)	∴	35 (7)	0.189
Combination therapy	∴	39.5 (17)	∴	65 (13)	0.103
*Outcomes and complications*					
Relapse of positive blood culture episode at least 14 days after the first	∴	7 (3)	∴	5 (1)	1.000
Length of stay (days)	40.0 [24.5–67.0]	∴	41.5 [17.5–86.5]	∴	0.706
14-day mortality	∴	9.3 (4)	∴	0 (0)	0.298
30-day mortality	∴	11.6 (5)	∴	5 (1)	0.655
In-hospital mortality	∴	16.3 (7)	∴	15 (3)	1.000

*Abbreviations* IQR: interquartile range; ICU: Intensive Care Unit

Comparison of the clinical characteristics of the patients who died during admission vs. those who survived (Table 4) showed non-survivors suffering more from diabetes (*p* = 0.025), having a higher Charlson Comorbidity index (*p* = 0.036), having been treated with antibiotic therapy in the previous seven days (*p* = 0.038), and having a longer time from admission to positive BC episode (*p* = 0.003) than survivors. No more statistically significant differences in terms of patient characteristics, microbiological findings, reason for admission, source of infection, severity of bacteremia, antibiotic management and complications were observed.

**Table 4:** Comparison of clinical characteristics of survivors vs. non-survivors during the course of admission (in-hospital mortality)

Feature	Non-survivors *n* = 10		Survivors *n* = 53		*p*-value
	Median [IQR]	% (*n*)	Median [IQR]	% (*n*)	Fisher’s exact/ Mann-Whitney
*Patient characteristics*					
Age	72.0 [67.5–74.8]	∴	61.0 [49.0–74.0]	∴	0.110
Male gender	∴	50 (5)	∴	62.3 (33)	0.500
Medical	∴	50 (5)	∴	45.3 (24)	1.000
Surgical	∴	20 (2)	∴	22.6 (12)	1.000
Critically ill	∴	30 (3)	∴	32.1 (17)	1.000
Diabetes	∴	50 (5)	∴	15.1 (8)	0.025 *
Cardiovascular disease	∴	60 (6)	∴	50.9 (27)	0.735
Chronic kidney disease	∴	30 (3)	∴	13.2 (7)	0.189
Chronic respiratory disease	∴	30 (3)	∴	9.4 (5)	0.106
Chronic liver disease	∴	10 (1)	∴	13.2 (7)	1.000
Solid neoplasm	∴	30 (3)	∴	15.1 (8)	0.360
Hematological neoplasm	∴	20 (2)	∴	17 (9)	1.000
Solid organ transplant recipient	∴	10 (1)	∴	9.4 (5)	1.000
Hematopoietic stem-cell transplantation	∴	10 (1)	∴	3.8 (2)	0.410
Immunosuppressive therapy	∴	20 (2)	∴	20.8 (11)	1.000
Long-term intravascular device	∴	90 (9)	∴	67.9 (36)	0.257
Charlson Comorbidity Index	5.5 [4.0-6.75]	∴	4.0 [2.0–6.0]	∴	0.036 *
Rectal colonization by multidrug resistant organism	∴	40 (4)	∴	37.7 (20)	1.000
Antibiotic therapy in the previous seven days	∴	80 (8)	∴	41.5 (22)	0.038 *
*Microbiological findings*					
Time from admission to positive blood culture episode (days)	37.5 [28.0-81.8]	∴	14.0 [2.0–28.0]	∴	0.003 *
At least 50% of blood cultures positive	∴	70 (7)	∴	67.9 (36)	1.000
*Stenotrophomonas maltophilia*	∴	60 (6)	∴	47.2 (25)	0.509
*Achromobacter* species	∴	10 (1)	∴	17 (9)	1.000
*Ochrobactrum* species	∴	0 (0)	∴	11.3 (6)	0.578
*Aeromonas* species	∴	0 (0)	∴	11.3 (6)	0.578
*Burkholderia* species	∴	20 (2)	∴	5.7 (3)	0.175
Other	∴	10 (1)	∴	15.1 (8)	1.000
Polymicrobial blood culture	∴	30 (3)	∴	43.4 (23)	0.504
*Reason for admission*					
Trauma	∴	10 (1)	∴	22.6 (12)	0.672
Respiratory failure	∴	10 (1)	∴	5.7 (3)	0.508
Cardiovascular failure	∴	0 (0)	∴	5.7 (3)	1.000
Liver failure	∴	0 (0)	∴	5.7 (3)	1.000
Renal failure	∴	0 (0)	∴	1.9 (1)	1.000
Sepsis	∴	30 (3)	∴	26.4 (14)	1.000
Surgery	∴	40 (4)	∴	22.6 (12)	0.259
Burn injury	∴	10 (1)	∴	5.7 (3)	0.508
Stroke	∴	0 (0)	∴	3.8 (2)	1.000
*Source of infection*					
Catheter-related	∴	30 (3)	∴	30.2 (16)	1.000
Primary	∴	10 (1)	∴	18.9 (10)	0.676
Respiratory tract	∴	10 (1)	∴	20.8 (11)	0.671
Urinary tract	∴	10 (1)	∴	0 (0)	0.159
Intra-abdominal	∴	20 (2)	∴	15.1 (8)	0.653
Soft tissue	∴	10 (1)	∴	11.3 (6)	1.000
*Severity of bloodstream infection*					
ICU admission	∴	10 (1)	∴	15.1 (8)	1.000
Pitt Bacteremia Score	1.5 [0.0–2.0]	∴	1.0 [0.0–2.0]	∴	1.000
Neutropenia	∴	20 (2)	∴	7.6 (4)	0.240
*Management*					
No treatment	∴	10 (1)	∴	3.8 (2)	0.410
Active antibiotic therapy within 48 h	∴	50 (5)	∴	56.6 (30)	0.739
*Targeted antibacterial therapy*					
Monotherapy	∴	30 (3)	∴	50.9 (27)	0.308
Combination therapy	∴	60 (6)	∴	45.3 (24)	0.498

*Abbreviations* IQR: interquartile range; ICU: Intensive Care Unit

**Fig. 1:**
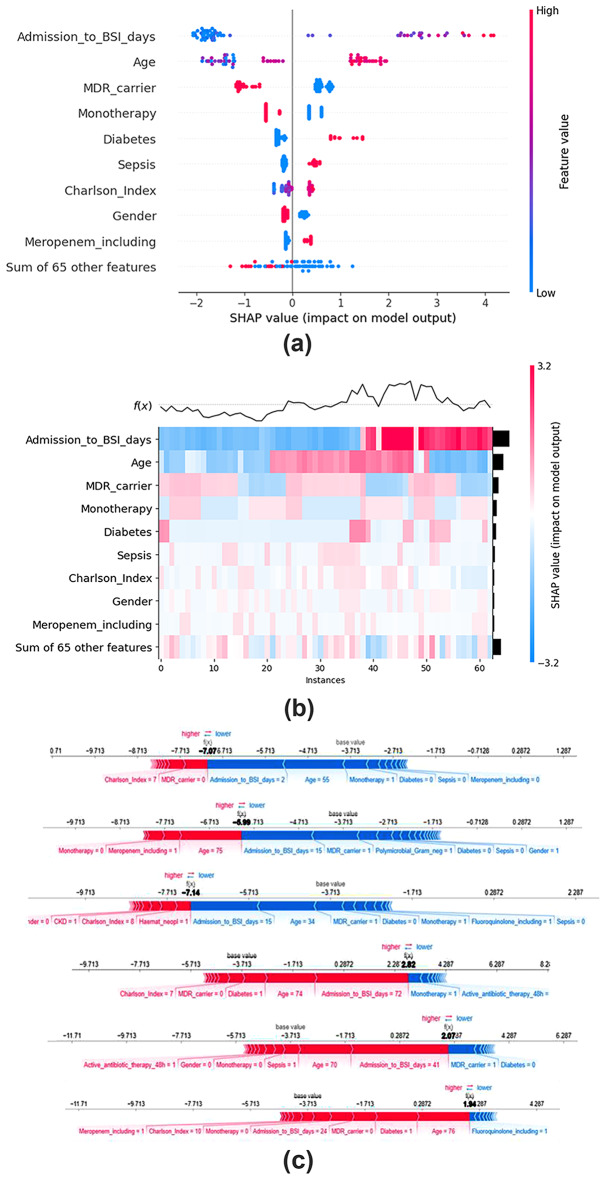
XGBoost explanatory model to rank and identify relative feature importance of factors associated with in-hospital mortality. **(a)** Beeswarm Plot of SHAP Values: Beeswarm plot illustrating the distribution of SHAP (SHapley Additive exPlanations) values for each feature in the dataset. Each dot represents a feature value for a specific patient, with the position along the x-axis indicating the magnitude of the SHAP value. The color of each dot indicates the corresponding feature value, providing insight into the relationship between feature values and their impact on model predictions. Features with wider distributions and greater dispersion of SHAP values suggest higher variability and importance in the model’s decision-making process. Redder dots on the right side of the plot indicate that being positive for that feature or experiencing higher values on it increases the predicted probability of death. Redder dots on the left side indicate the opposite (lower chances of dying). **(b)** Heatmap of SHAP Values: Heatmap illustrating the SHAP (SHapley Additive exPlanations) values for the top features impacting the model’s mortality prediction scores. Each row corresponds to a feature in the dataset, and each column represents a patient sample. Patient samples are ordered using hierarchical clustering by their explanation similarity, resulting in samples with closer model outputs for the same reason getting grouped together. The color intensity indicates the magnitude and direction of the feature’s impact on the model output: red indicates positive impact (increasing the predicted likelihood of death), while blue indicates negative impact (decreasing the predicted likelihood of death). A feature’s importance can be inferred from the range and variability of its SHAP values across samples. Features with higher absolute SHAP values exert a greater influence on the model predictions. Only the top nine features are herein individually depicted. The output of the model is shown above the heatmap matrix as a line plot centered around the explanation’s base value and the global importance of each model input shown as a bar plot on the right hand side of the plot. **(c)** Force plots with individual patient examples, breaking down the contribution of each feature to the prediction of a given patient (three random patients with a high/low predicted score shown). Scores are on a log odds scale. Probabilities can be easily inferred as probability = exp(log-odds)/(1 + exp(log-odds))

The XGBoost model used to rank features based on their relative importance based on their contribution towards mortality risk patient stratification suggested the most informative features to be the time from admission to the positive BC episode (a high cardinality feature, which inherently increases its importance), older age, diabetes, admission due to sepsis, higher Charlson Comorbidity Index, being treated with combination therapy, and being treated with a meropenem-including regimen. Additionally, the model underscored that male patients and those colonized by multidrug-resistant bacteria were less likely to die compared to female patients and non-carriers under surveillance, respectively (Fig. 1).

## Discussion

This study offers a contemporary insight into the clinical–microbiological features of positive BC episodes sustained by NFGNB other than *Pseudomonas* and *Acinetobacter* species in a cohort of patients admitted in a reference center of Northern Italy encompassing all major medical and surgical specialties and with multiple challenges in the area of antibiotic resistance [38]. Its findings revealed that positive BC episodes sustained by NFGNB other than *Pseudomonas* and *Acinetobacter* species were polymicrobial and microbiologically significant in a remarkable proportion of the samples. *Stenotrophomonas maltophilia* was the most detected species and, together with *Achromobacter* species, exhibited the most complex resistance phenotype. Clinically, positive BC episodes were more frequent in patients with a long-term intravascular device and had BSI criteria in the vast majority of cases. In contrast, the degree of severity of bacteremia was quite low, as were the associated mortality rates. Time from admission to the positive BC episode, older age, diabetes, admission due to sepsis, higher Charlson Comorbidity Index, being treated with combination therapy and meropenem-inclusive regimen emerged as factors associated with in-hospital mortality.

Knowledge of the clinical significance of NFGNB other than *Pseudomonas* and *Acinetobacter* species is currently very limited [6]. Our study only partially assessed the impact of these species given the high proportion of polymicrobial samples. However, our results reported a high correspondence between positive BC episode, ≥ 50% positive/overall BC samples ratio, BSI criteria and targeted treatment prescription, adding some evidence in favor of the likely pathogenic potential of these species.

Our study also confirmed the epidemiological and clinical relevance of *Stenotrophomonas maltophilia* within the less common NFGNB [6]. A substantial literature already exists on this species and associated infections [18, 39–47] so much so that it is the only one to have been included in the Infectious Diseases Society of America treatment guidelines [36]. Our study also highlighted that resistance to sulfamethoxazole/trimethoprim and cefiderocol remains uncommon as already highlighted in recent studies [13, 48].

Despite the limited sample size, our study showed the complex phenotype of *Achromobacter* species exhibiting low susceptibility to piperacillin/tazobactam, third-generation cephalosporins, carbapenems, and fluoroquinolones. These findings are not consistent with the results of a Spanish study on patients suffering from *Achromobacter xylosoxidans* bacteremia that showed high susceptibility to meropenem using EUCAST breakpoints for *Pseudomonas aeruginosa* [49], highlighting *Achromobacter* potential for innate resistance to all antibiotics [18]. Of note, high values of inhibition zone diameter with cefiderocol were observed among the isolates included in our study, as also previously described [14]. This might highlight cefiderocol as promising treatment of severe *Achromobacter* species infections, although clinical evidence on in vivo activity is still limited.

The opportunistic pathogenic nature of the less common NFGNB is highlighted in the literature by the fact that they mainly cause nosocomial infections in an increasingly common population with risk factors such as neoplasia or chronic organ disease, prolonged hospitalizations and the need for systemic therapies, including antibiotics, through long-term intravascular devices [18]. This is consistent with the results of our study which identified the presence of a long-term intravascular device as the most frequent clinical feature among patients and the main source of BSI. On the other hand, although our study showed quite low BSI severity indices and consequently low mortality rates, evidence available so far shows different data according to pathogen and cohort of patients considered [6]. Mortality rates among adult patients suffering from *Stenotrophomonas maltophilia* BSIs ranged from 32 to 54.8% [50–52], while for those sustained by *Achromobacter* species from 20 to 27% [53, 54]. Similar rates have been reported in *Elizabethkingia* species BSIs [55–57], while lower rates have been reported in *Chryseobacterium* species [58].

Perhaps not surprising, in our study patients who did not survive admission were older, suffered from more baseline comorbidities, were admitted due to sepsis, and experienced both longer time from admission to the positive BC episode and more broad-spectrum antibiotic therapy. Therefore, patient’s general condition prevailed over etiology in patients suffering from less common NFGNB BSIs. The relevance of comorbidities in predicting mortality was well highlighted in an Australian population study over the period 2000–2019 [53]. In a recent meta-analysis on patients with *Stenotrophomonas maltophilia* bloodstream infection, poor outcomes were associated with infection severity (ICU admission, septic shock, need for mechanical ventilation), comorbidities (indwelling central venous catheter, neutropenia, hematological neoplasms, chronic kidney disease) and antibiotic prescription (inappropriate antimicrobial therapy and prior antibiotic use) [59]. However, our finding differs significantly from a recent multicenter study conducted in Northern Italy on patients suffering from NFGNB BSI (including *Pseudomonas aeruginosa* and *Acinetobacter baumannii* complex) that identified older age, septic shock and *Acinetobacter baumannii* complex etiology as predictors of 30-day mortality [11]. The strength of this study is the collection of data from a surveillance study on a group of bacteria sporadically reported in the literature and investigating their pathogenic potential. Some limitations should be also acknowledged, including its retrospective nature with possible confounders and potential bias not considered and the fact that it was conducted in a single center. The narrow nature of our findings, due to the limited sample size, does not allow generalized conclusions to be drawn.

In conclusion, this study investigated the clinical and microbiological findings of positive BC episodes caused by NFGNB other than *Pseudomonas* and *Acinetobacter* species. Positive BC episodes were predominantly sustained by *Stenotrophomonas maltophilia* and *Achromobacter* species, having BSI criteria in the vast majority of cases. *Achromobacter* species isolates showed the most complex resistance phenotype and from the in vitro results, cefiderocol appeared to be a possible option for treating infections caused by these multidrug resistance strains. Long-term intravascular device and respiratory tract were the main sources of infection. No differences in patient comorbidities, infection severity and outcomes were observed on the basis of the percentage of positive BC samples, providing an argument in favor of those who consider these species to have substantial pathogenic potential. The longer time from admission to positive BC episode, older age, diabetes, admission due to sepsis, higher Charlson Comorbidity Index, being treated with combination therapy and meropenem-including regimen emerged as the strongest predictors of in-hospital mortality. This might highlight how these species may have more room in prolonged hospitalisation and at the end of life for patients with chronic organ diseases. Further studies are needed to validate our results, notwithstanding the need for continuous surveillance of these species and their antimicrobial susceptibility profiles.

## Data Availability

Data is provided within the manuscript or supplementary information files.
